# Economic Value, Relative Vulnerability and Honeybee Colony Supply–Demand Imbalance in Chinese Agriculture, 2010–2024

**DOI:** 10.3390/insects17070673

**Published:** 2026-06-27

**Authors:** Yongpeng Chen, Zhijun Zhao, Shemei Zhang

**Affiliations:** 1 School of Economics Management, Inner Mongolia Normal University, No. 81 Zhaowuda Road, Saihan District, Hohhot 010022, China; chenyp@mail.bnu.edu.cn; 2 Institute of Agricultural Economics and Development, Chinese Academy of Agricultural Sciences, No. 12 Zhongguancun South Street, Beijing 100081, China; zhaozhijun@caas.cn; 3 College of Management, Sichuan Agricultural University, No. 211 Huimin Road, Wenjiang District, Chengdu 611130, China

**Keywords:** honeybee pollination, pollination service value, relative vulnerability, colony demand, pollination service capacity, supply–demand gap, high-value agriculture, China

## Abstract

Managed honeybee colonies contribute to agriculture through multiple ecosystem services. However, the contribution of honeybees is often assessed through bee products rather than through the crop pollination services they provide. This study quantified the economic value, relative vulnerability and supply–demand imbalance of honeybee pollination in Chinese agriculture from 2010 to 2024. Using crop production, gross production value, harvested area and managed colony data, combined with crop-specific pollination-dependence coefficients, we estimated pollination function quantity, service value, relative vulnerability, theoretical colony demand and supply gaps. Benchmark pollination service value increased from US$114.27 billion in 2010 to US$247.02 billion in 2024, while pollination-attributable output increased from 203.92 million tonnes to 274.73 million tonnes. In 2024, 36.94% of selected crop value was exposed to pollination dependence, with vegetables and fruits contributing more than 93% of total benchmark value. Effective honeybee supply was estimated at 5.73 million colonies in 2024, resulting in a substantial supply deficit of 68.00 million colonies and a demand-to-effective-supply ratio of 12.88 under the benchmark scenario. Despite sensitivity to colony-use assumptions, the demand–supply gap remained persistent across alternative specifications. These results show that China’s high-value agriculture increasingly depends on pollination services while managed pollination capacity remains far below theoretical demand.

## Simple Summary

Many fruits, vegetables and oil crops need bees and other insects to produce good harvests. In China, these crops are becoming more valuable, but it is unclear whether there are enough managed honeybee colonies to provide the pollination they need. This study examined China’s crop production, crop value, crop area and managed honeybee colony numbers from 2010 to 2024. We found that the value of crop production linked to pollination more than doubled, from 114.27 billion United States dollars in 2010 to 247.02 billion United States dollars in 2024. In 2024, more than one-third of the value of the selected crops depended on pollination, and vegetables and fruits accounted for most of this value. However, the number of honeybee colonies needed for crop pollination was far higher than the number actually available. These findings show that bees should be viewed not only as producers of honey, but also as an important support system for high-value agriculture. Better pollination services, safer pesticide use and improved bee colony placement are needed.

## 1. Introduction

Pollination is a fundamental ecosystem service supporting agricultural production, food quality and the stability of many high-value crop systems. Although major staple grains are largely wind- or self-pollinated, a substantial share of fruits, vegetables, oil crops, nuts and seed crops depends partly or strongly on animal-mediated pollination [1]. Pollinators contribute not only to fruit set and seed formation, but also to yield stability, product quality and marketable output. Global assessments have shown that animal pollination contributes substantially to agricultural production value and that pollinator decline may expose food systems to economic, nutritional and health risks [1,2,3,4]. Consequently, pollination should be understood not only as an ecological process, but also as a measurable biological input embedded in modern agricultural production.

Within the ecosystem-services framework [5], honeybee colonies contribute to agriculture and human welfare through multiple service categories. Honey, royal jelly, beeswax and other bee products represent provisioning services, whereas crop pollination represents a regulating service because it supports crop reproduction and stabilizes agricultural output [6]. Honeybee colonies may also contribute to cultural ecosystem services through education, traditional knowledge, recreation, landscape esthetics and public awareness of biodiversity. In addition, by supporting plant reproduction in agricultural landscapes, pollinators are linked to broader agroecosystem functions. Because the present study focuses on crop pollination services and does not directly quantify cultural services, supporting functions or other non-market contributions, the estimated value should be interpreted as a conservative, lower-bound estimate of the broader ecosystem-service contributions of honeybee colonies.

Managed honeybees are especially important because they are among the most widely mobilized pollinators in commercial agriculture. Unlike most wild pollinators, managed honeybee colonies can be transported, concentrated and deployed during crop flowering periods, making them particularly important for large-scale fruit, vegetable, cucurbit and oilseed production [6,7]. However, the capacity of honeybees to provide crop pollination services depends not only on the number of colonies, but also on colony strength, beekeeper mobility, floral-resource continuity, pesticide exposure, landscape context, pollination-service incentives and coordination between growers and beekeepers [8,9,10,11,12]. Therefore, the observed stock of managed colonies does not necessarily represent the effective supply of crop pollination services.

China provides an important case for examining this issue. Over recent decades, Chinese agriculture has shifted from a production system primarily oriented toward staple food security to a more diversified system involving fruits, vegetables, oil crops, nuts and other high-value commodities. Many of these crops are pollination-dependent or pollination-responsive. As their harvested area and production value expand, the economic exposure of Chinese agriculture to pollination services is likely to increase. At the same time, China’s beekeeping sector has traditionally been treated mainly as a bee-product industry, with honey, royal jelly, beeswax and other products forming the main market focus. Pollination services for cash and industrial crops remain less institutionalized, and growers in many regions still rely on partial self-pollination, unmanaged insects, manual pollination or informal beekeeper access rather than formal pollination contracts.

Existing Chinese studies have established the importance of pollination valuation. Liu et al. (2011) estimated the economic value of bee pollination for major crops in China and provided an early national benchmark for the role of honeybees in agricultural production [13]. Ouyang et al. (2019) broadened the perspective to insect pollination, summarized crop-level dependence coefficients and evaluated pollination function and service value in Chinese agricultural ecosystems [14]. These studies demonstrated that pollination contributes substantial economic value to Chinese agriculture. However, three gaps remain. First, existing national estimates are mostly static or based on earlier benchmark years, and therefore do not fully capture the recent evolution of China’s crop structure. Second, most valuation studies emphasize absolute economic value but pay less attention to relative vulnerability, namely the share of crop production value exposed to pollination dependence. Third, economic valuation alone cannot reveal whether managed honeybee colony supply is sufficient to meet the expanding demand for crop pollination. A crop system may generate high pollination value while facing insufficient colony supply, creating a structural imbalance between pollination demand and managed pollinator capacity. International studies have also shown that managed honeybee stocks may grow more slowly than crop-level pollination demand, creating supply–demand mismatches in agricultural systems [15,16,17,18].

To address these gaps, this study develops a national macro-level assessment of pollination service value, relative vulnerability and honeybee colony supply–demand imbalance in Chinese agriculture from 2010 to 2024. Using FAOSTAT crop production, gross production value, harvested area and managed honeybee colony data, combined with crop-specific pollination-dependence coefficients, we estimate pollination function quantity, pollination service value, relative vulnerability, theoretical colony demand, pollination service capacity and supply gaps. The remainder of the paper is organized as follows. Section 2 describes the background on China’s crop production structure, the data sources, crop classification, pollination-dependence coefficients and model equations. Section 3 reports national trends, category-level patterns and crop-level priorities in pollination value, vulnerability and colony demand. Section 4 discusses the implications for ecosystem-service valuation, commercial pollination markets and agricultural risk governance. Section 5 concludes by summarizing the main findings and policy implications.

## 2. Materials and Methods

### 2.1. Data Sources, Crop Selection and Analytical Crop Categories

This study constructed a national crop-level panel for China from 2010 to 2024. Crop harvested area, production quantity, yield and managed honeybee colony stock were obtained from the FAOSTAT Crops and Livestock Products domain. Crop gross production value was obtained from the FAOSTAT Value of Agricultural Production domain and was used as the basis for economic valuation.

The analysis included 44 FAOSTAT crop items relevant to pollination-dependent or pollination-responsive agriculture. These crops were grouped into four mutually exclusive analytical categories: staple and field food crops, fruit and nut crops, vegetable and cucurbit crops, and cash and industrial crops (Table 1). Each crop item was assigned to only one category to avoid double-counting. Staple and field food crops refer to cereals, pseudocereals, pulses and selected field crops primarily associated with food-security production or broad field-crop systems. Fruit and nut crops include pome fruits, stone fruits, citrus fruits, tropical fruits, berries and nut crops. Vegetable and cucurbit crops include cucurbits and fruit vegetables commonly produced for fresh consumption or horticultural markets. Cash and industrial crops refer to oil, fibre, beverage and other market-oriented crops mainly grown for processing, industrial use or commercial sale. All valuation and colony-demand calculations were first conducted at the FAOSTAT crop-item level. The full crop-item classification, dependence class, dependence coefficients and recommended colony density are provided in Table S1.

The selected crop panel covers crop groups with contrasting levels of pollination dependence and harvested-area characteristics. Cucurbits such as cucumbers and gherkins, watermelons, cantaloupes and pumpkins were classified as essential-dependence crops. Several fruit and nut crops, including apples, pears, peaches and nectarines, plums and sloes, citrus-type fruits, almonds and kiwi fruit, were classified as moderate, high or essential dependence crops. By contrast, staple and field food crops such as rice, beans, buckwheat and soybeans showed lower or more heterogeneous dependence levels. Cash and industrial crops, including rape or colza seed, sesame seed, seed cotton, groundnuts and coffee, differed in both dependence coefficients and harvested area. This classification provides the crop background for interpreting the subsequent differences between pollination service value and colony demand.

### 2.2. China’s Pollination-Responsive Crop-Area Transition and Managed Honeybee Supply

Within the selected 44-crop FAOSTAT panel, China’s harvested-area structure shifted toward crop groups more closely associated with managed pollination demand. From 2010 to 2024, staple and field food crop harvested area remained almost stable, while cash and industrial crops area decreased from 17.66 million ha to 16.22 million ha (−8.2%). By contrast, fruit and nut crop harvested area increased from 10.15 million ha to 12.37 million ha (+21.8%), and vegetable and cucurbit crop harvested area increased from 7.69 million ha to 8.39 million ha (+9.1%). Because many fruit, vegetable, cucurbit and oilseed crops benefit from insect-mediated pollination, this crop-area transition indicates that potential demand for managed pollination services expanded more rapidly in pollination-responsive crop groups than in staple-oriented crop groups.

However, managed honeybee colony growth was much more limited. FAOSTAT data show that China’s managed honeybee colony stock increased from 8.90 million colonies in 2010 to 9.54 million colonies in 2024, equivalent to an increase of 0.64 million colonies, or 7.2%, over the study period. Colony stock is also not equivalent to effective crop pollination supply, because not all colonies are strong, mobile, geographically matched to crop flowering, or economically available for field pollination. This contrast between expanding pollination-responsive crop area and limited managed-colony growth motivates the following analysis of pollination value, colony demand and honeybee supply–demand gaps.

### 2.3. Pollination Dependence Coefficient

Crop-specific pollination dependence was assigned using dependence ranges reported in Chinese pollination valuation studies [1,14]. For each crop *i*, three coefficients were used: $D_{low,i}$, $D_{mid,i}$ and $D_{high,i}$. The midpoint coefficient was used as the benchmark estimate, while the lower and upper coefficients were used to construct uncertainty ranges. Five dependence classes were defined: no dependence (*D* = 0), low dependence (0< *D* <0.10), moderate dependence (0.10 ≤ *D* < 0.40), high dependence (0.40 ≤ *D* < 0.90), and essential dependence (0.90 ≤ *D* ≤ 1.00). The corresponding $D_{low}$, $D_{mid}$, and $D_{high}$ values were 0.00–0.00–0.00, 0.00–0.05–0.10, 0.10–0.25–0.40, 0.40–0.65–0.90, and 0.90–0.95–1.00, respectively. Crops with unclear dependence were retained in the crop list but assigned zero dependence in the aggregate calculations unless otherwise specified. The crop-specific dependence classes and the corresponding *D* values are listed in Table S1.

### 2.4. Estimating Pollination Function, Service Value and Relative Vulnerability

Pollination function quantity was used to measure the physical crop output attributable to insect pollination. For crop *i* in year *t* is calculated as:

$$PF_{it}^{k}=Y_{it}D_{i}^{k}$$

where $PF_{it}^{k}$ is the pollination function quantity, $Y_{it}$ is crop production quantity, $D_{i}^{k}$ is the pollination-dependence coefficient, and k∈{low,mid,high}. National annual pollination function quantity was obtained by summing across crops.

Pollination service value was calculated by multiplying crop gross production value by the corresponding dependence coefficient:

$$PV_{it}^{k}=GPV_{it}D_{i}^{k}$$

where $PV_{it}^{k}$ is pollination service value and $GPV_{it}\;$is crop gross production value. The national annual value is:

$$PV_{t}^{k}=\sum_{i}GPV_{it}D_{i}^{k}$$

Current-price values were reported in million US dollars. This dependence-ratio approach follows previous pollination valuation studies, while recognizing that such estimates represent production-value exposure rather than marginal ecosystem-service values [19,20,21].

Relative vulnerability was used to measure the proportion of crop production value exposed to pollination dependence. At the national level, it is calculated as:

$$RV_{t}^{k}=\frac{\sum_{i}GPV_{it}D_{i}^{k}}{\sum_{i}GPV_{it}}$$

For crop category *c*, category-level relative vulnerability is calculated as:

$$RV_{ct}^{k}=\frac{\sum_{i\in c}GPV_{it}D_{i}^{k}}{\sum_{i\in c}GPV_{it}}$$

Thus, *RV* represents a value-weighted measure of pollination exposure rather than a simple average of crop-level dependence coefficients.

### 2.5. Estimating Honeybee Pollination Demand and Colony Supply Gaps

Honeybee pollination demand was estimated using crop harvested area and recommended colony density. For crop *i* in year *t*, theoretical colony demand [13] is calculated as:

$$HPD_{it}=A_{it}RCD_{i}$$

where $A_{it}$ is the harvested area and $RCD_{i}$ is the recommended number of colonies per hectare. Because crop-specific technical standards are not consistently available for all FAOSTAT crop items, a benchmark density scheme was used: fruit and nut crops were assigned 4.4 colonies/ha; vegetable and cucurbit crops 4.3 colonies/ha; cash and industrial crops 2.5 colonies/ha; legumes and selected staple and field food crops 1.4 colonies/ha. The recommended colony density assigned to each FAOSTAT crop item is reported in Table S1.

The raw national colony demand [13] is calculated as:

$$HPD_{t}^{raw}=\sum_{i}A_{it}RCD_{i}$$

Following previous honeybee pollination-demand assessments [18], raw colony demand is adjusted using an annual colony-use factor, m:

$$HPD_{t}^{adj}=\frac{HPD_{t}^{raw}}{m}$$

where m represents the number of effective crop pollination uses that one managed honeybee colony can provide within a year in the national accounting framework. The benchmark value was set to *m* = 2, following the literature-based adjustment used in previous assessments. In China, stationary and migratory beekeeping systems coexist, and migratory colonies may follow multiple flowering periods within a year. Therefore, sensitivity analyses were conducted using *m* = 4 and *m* = 6 to represent more intensive annual colony-use scenarios.

The annual stock of managed honeybee colonies, $HC_{t}$, is used as nominal colony supply. Pollination service capacity, supply gap and demand-to-supply ratio are calculated as:

$$PSC_{t}^{nominal}=\frac{HC_{t}}{HPD_{t}^{adj}}$$

$$Gap_{t}^{nominal}=HPD_{t}^{adj}-HC_{t}$$

$$DSR_{t}^{nominal}=\frac{HPD_{t}^{adj}}{HC_{t}}$$

To account for the fact that not all managed colonies are effectively available for crop pollination, nominal colony stock is further adjusted using an effective availability coefficient:

$$HC_{t}^{eff}=HC_{t}\theta$$

where *θ* = 0.6. Effective supply gap, effective pollination service capacity and effective demand-to-supply ratio are calculated as:

$$Gap_{t}^{eff}=HPD_{t}^{adj}-HC_{t}^{eff}$$

$$PSC_{t}^{eff}=\frac{HC_{t}^{eff}}{HPD_{t}^{adj}}$$

$$DSR_{t}^{eff}=\frac{HPD_{t}^{adj}}{HC_{t}^{eff}}$$

The coefficient *θ* was used as a benchmark correction to distinguish nominal colony stock from effective pollination-service capacity. It should not be interpreted as an empirically observed national share of colonies used for commercial pollination.

### 2.6. Aggregation, Crop Prioritization and Visualization

Category-level contributions to pollination service value and colony demand are calculated as:

$$SharePV_{ct}=\frac{PV_{ct}^{mid}}{PV_{t}^{mid}}$$

$$ShareHPD_{ct}=\frac{HPD_{ct}^{adj}}{HPD_{t}^{adj}}$$

The top ten crops by benchmark pollination service value and the top ten crops by adjusted colony demand were identified for 2024. Crop-level value, vulnerability and colony-demand rankings were compared to identify priority crops.

To assess temporal divergence between demand and supply, adjusted pollination demand and managed colony stock are normalized to 100 in 2010:

$$IndexHPD_{t}=\frac{HPD_{t}^{adj}}{HPD_{2010}^{adj}}\times 100$$

$$IndexHC_{t}=\frac{HC_{t}}{HC_{2010}}\times 100$$

The scissors-difference index is calculated as:

$$SD_{t}=IndexHPD_{t}-IndexHC_{t}$$

All calculations were implemented in R [22]. Data processing used dplyr [23], tidyr [24] and stringr [25]; Excel outputs were generated using openxlsx [26]; and figures were produced using ggplot2 [27].

## 3. Results

### 3.1. National Trends in Pollination Function, Service Value and Vulnerability

Pollination function quantity increased substantially during 2010–2024. Under the benchmark dependence coefficient, pollination-attributable crop output increased from 203.92 million tonnes in 2010 to 274.73 million tonnes in 2024, representing a 34.7% increase. In 2024, the uncertainty range based on $D_{low}$, $D_{mid}$, and $D_{high}$ was 208.13–341.33 million tonnes.

Pollination service value increased even more strongly. The benchmark value rose from US$114.27 billion in 2010 to US$247.02 billion in 2024, more than doubling over the study period. The lower- and upper-bound estimates increased from US$81.09–147.45 billion in 2010 to US$177.38–316.65 billion in 2024. Over the same period, the gross production value of the selected crops increased from US$318.23 billion to US$668.73 billion. Thus, the increase in pollination service value reflected both the expansion of crop production value and the growing importance of pollination-responsive crops within China’s agricultural value structure.

**Figure 1:**
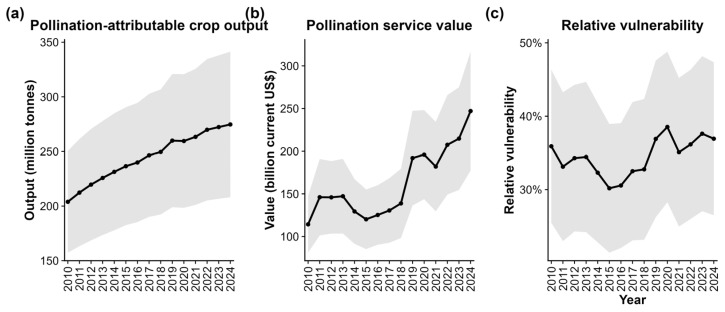
National trends in pollination function quantity, service value and relative vulnerability in Chinese agriculture, 2010–2024. (**a**) Pollination-attributable crop output; (**b**) pollination service value; (**c**) relative vulnerability. Solid lines represent benchmark estimates based on $D_{mid}$ or ${RV}_{mid}$, and shaded areas represent uncertainty ranges based on $D_{low}$–$D_{high}$ or ${RV}_{low}$–${RV}_{high}$. Pollination service value is reported in current US dollars.

Relative vulnerability remained consistently high. National ${RV}_{mid}$ increased from 35.91% in 2010 to 36.94% in 2024, with a 2024 uncertainty range of 26.53–47.35%. This means that, under the benchmark dependence coefficient, more than one-third of the gross production value of the selected crops was exposed to pollination dependence in 2024. Although absolute pollination value increased sharply, RV changed only moderately, suggesting that the rise in value was driven mainly by the expansion of crop value rather than by a large shift in the aggregate dependence structure. These national temporal trends in pollination-attributable output, service value and relative vulnerability are summarized in Figure 1.

### 3.2. Category-Level Concentration of Pollination Value and Vulnerability

Pollination service value was concentrated mainly in vegetable and cucurbit crops and fruit and nut crops. In 2024, vegetable and cucurbit crops contributed US$121.48 billion, accounting for 49.2% of total benchmark pollination service value. Fruit and nut crops contributed US$108.55 billion, accounting for 43.9%. Together, vegetable and cucurbit crops and fruit and nut crops accounted for approximately 93.1% of total pollination service value. Cash and industrial crops contributed US$11.54 billion (4.7%), while staple and field food crops contributed US$5.45 billion (2.2%).

**Figure 2:**
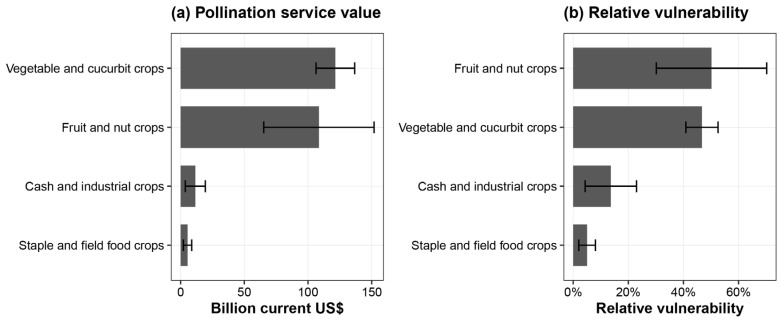
Category-level concentration of pollination service value and relative vulnerability in 2024. (**a**) Pollination service value by crop category; (**b**) relative vulnerability by crop category. In panel (**a**), bars represent benchmark estimates based on $D_{mid}$, and error bars represent uncertainty ranges based on $D_{low}$ and $D_{high}$ In panel (**b**), bars represent ${RV}_{mid}$, and error bars represent the${\;RV}_{low}$–${RV}_{high}$ range.

Fruit and nut crops showed the highest proportional exposure to pollination dependence. Fruit and nut crops had the highest ${RV}_{mid}$, at 50.20%, followed by vegetable and cucurbit crops at 46.73%. Cash and industrial crops had a much lower ${RV}_{mid}$, at 13.66%, while staple and field food crops showed the lowest vulnerability, at 5.05%. Therefore, vegetable crops represented the largest absolute economic value at stake, whereas fruit crops were more exposed in proportional terms. This distinction indicates that pollination-related risk cannot be fully assessed using economic value alone. Figure 2 visualizes this contrast between absolute pollination service value and proportional exposure across crop categories.

### 3.3. Honeybee Pollination Demand, Colony Supply and Structural Gap

Adjusted honeybee pollination demand substantially exceeded managed colony supply throughout the study period. Under the benchmark annual colony-use factor (m = 2), adjusted demand increased from 68.06 million colonies in 2010 to 73.73 million colonies in 2024. Over the same period, the observed managed honeybee colony stock increased from 8.90 million to 9.54 million colonies. The nominal supply gap therefore increased from 59.16 million colonies in 2010 to 64.19 million colonies in 2024, and the nominal demand-to-supply ratio remained above seven, reaching 7.73 in 2024.

After applying the effective availability coefficient, effective colony supply was estimated at 5.34 million colonies in 2010 and 5.73 million colonies in 2024. Under the benchmark m = 2 scenario, the effective supply gap increased from 62.72 million colonies to 68.00 million colonies, and the effective demand-to-supply ratio reached 12.88 in 2024. Effective pollination service capacity remained below 8% throughout the study period, declining slightly from 7.84% in 2010 to 7.77% in 2024. These results indicate that the managed colony stock was far below the level required to meet theoretical crop pollination demand under the benchmark annual colony-use assumption.

Because the annual colony-use factor is a key assumption, we further assessed the sensitivity of the 2024 demand estimate to higher colony-use scenarios. When the colony-use factor was increased from m = 2 to m = 4 and m = 6, adjusted demand declined from 73.73 million colonies to 36.87 million and 24.58 million colonies, respectively. However, these values still exceeded the effective managed honeybee supply of 5.73 million colonies, producing demand-to-effective-supply ratios of 6.44 and 4.29 under the m = 4 and m = 6 scenarios. Thus, the estimated gap is sensitive to the annual colony-use assumption, but the conclusion that effective managed honeybee supply is insufficient remains unchanged.

**Figure 3:**
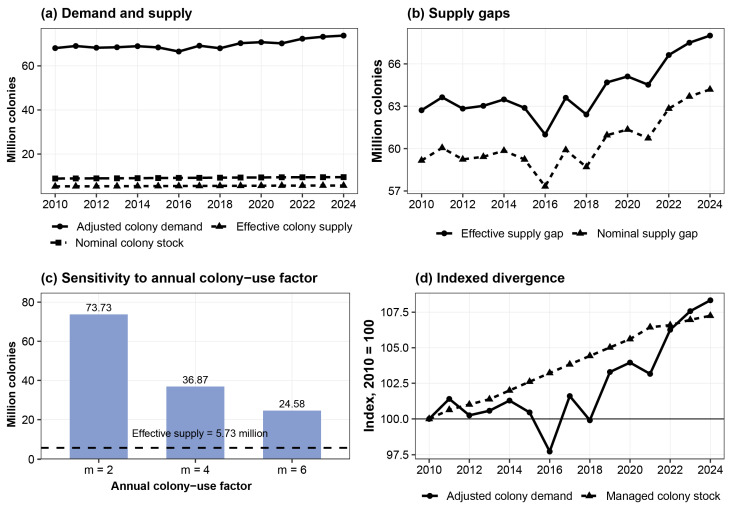
Honeybee pollination demand, managed colony supply and structural supply gap in China, 2010–2024. (**a**) Benchmark adjusted honeybee pollination demand (m = 2), nominal managed colony stock and effective colony supply; (**b**) nominal and effective supply gaps under the benchmark scenario; (**c**) sensitivity of 2024 adjusted colony demand to annual colony-use factors m = 2, m = 4 and m = 6, with the dashed line indicating effective managed colony supply; (**d**) indexed divergence between benchmark adjusted demand and managed colony stock, with 2010 normalized to 100. Effective supply was calculated using θ = 0.60.

The indexed comparison further showed that demand and supply developed only weakly asynchronously. When both indicators were normalized to 100 in 2010, adjusted pollination demand reached 108.34 in 2024, while managed colony supply reached 107.25. Although the growth-rate difference was modest, the absolute gap was already very large in 2010 and widened by 2024. Thus, the main issue is not a short-term fluctuation, but a persistent structural shortage of managed pollination capacity. The scale and persistence of this supply–demand imbalance are shown in Figure 3, which reports demand, supply, supply gaps, sensitivity to alternative annual colony-use factors and indexed divergence.

### 3.4. Crop-Level Priorities: Value, Vulnerability and Colony Demand

The crop-level rankings showed that pollination value and colony demand identify different dimensions of risk. In 2024, the top ten crops accounted for 83.4% of total benchmark pollination service value. The largest contributor was cucumbers and gherkins (US$59.93 billion), followed by apples (US$52.67 billion) and watermelons (US$38.42 billion). Other major contributors included pears (US$11.26 billion), tangerines, mandarins and clementines (US$9.73 billion), peaches and nectarines (US$8.66 billion), cantaloupes and other melons (US$8.27 billion), eggplants (US$6.14 billion), plums and sloes (US$5.54 billion) and soybeans (US$5.34 billion).

**Figure 4:**
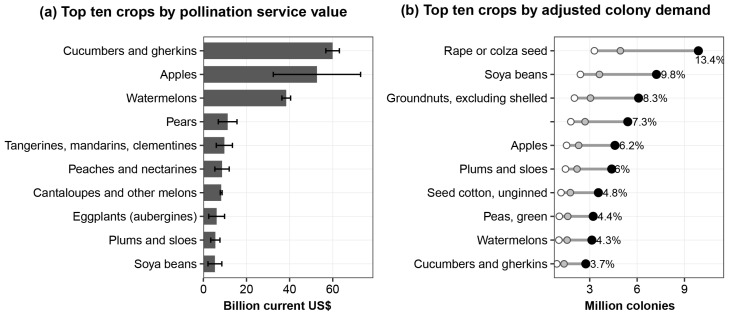
Crop-level concentration of pollination service value and adjusted honeybee colony demand in 2024. (**a**) Top ten crops by benchmark pollination service value. Bars represent benchmark estimates based on $D_{mid}$, and error bars represent the uncertainty range based on $D_{low}$ and $D_{high}$. (**b**) Top ten crops by adjusted honeybee colony demand. Black solid points represent benchmark adjusted colony demand under m = 2, grey solid points represent adjusted colony demand under m = 4, and open points represent adjusted colony demand under m = 6. Horizontal lines connect the three scenario values and indicate the sensitivity range under alternative annual colony-use factors. The rankings show that economic exposure and physical colony requirements identify different crop priorities.

The crop ranking for colony demand was different. In 2024, the top ten crops accounted for 68.2% of total adjusted colony demand. The largest contributor was rape or colza seed, requiring 9.88 million colonies (13.4% of total demand), followed by soybeans (7.23 million colonies, 9.8%), groundnuts (6.09 million colonies, 8.3%), tangerines, mandarins and clementines (5.41 million colonies, 7.3%) and apples (4.60 million colonies, 6.2%). Other major sources of colony demand included plums and sloes, seed cotton, green peas, watermelons, and cucumbers and gherkins. The difference between the two rankings is presented in Figure 4, showing that economic exposure and physical colony requirements do not identify the same set of priority crops.

**Figure 5:**
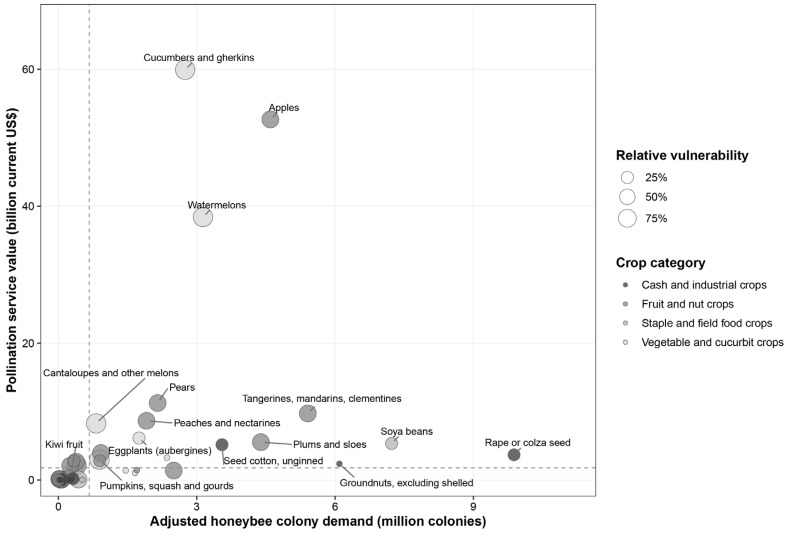
Crop priority matrix based on pollination service value, relative vulnerability and colony demand in 2024. Each point represents a crop. The horizontal axis shows adjusted honeybee colony demand, the vertical axis shows benchmark pollination service value, point size represents relative vulnerability, and grey shade represents crop category. Crops with higher values on both axes indicate greater combined economic exposure and colony-demand pressure.

Combining value, vulnerability and colony demand identifies three priority crop groups. The first group includes crops with both high economic value and high pollination dependence, such as cucumbers and gherkins, watermelons, cantaloupes and other melons, apples, pears, and tangerines, mandarins and clementines. These crops represent major economic exposure to pollination disruption. The second group includes crops with high colony demand but relatively lower proportional vulnerability or lower pollination value, such as rape or colza seed, soybeans, groundnuts, and seed cotton. These crops place strong pressure on managed colony allocation because of their extensive harvested area. The third group includes highly dependent but smaller-scale crops, such as kiwi fruit and pumpkins, squash and gourds, which may require targeted regional management despite their smaller national value contribution. To integrate these dimensions, Figure 5 plots crop-level pollination value against adjusted colony demand, while representing relative vulnerability and crop category through point size and grey shade.

## 4. Discussion

### 4.1. Pollination as a Growing Economic Input in Chinese Agriculture

This study shows that pollination services have become an increasingly important biological input in Chinese agriculture. The benchmark pollination service value increased from US$114.27 billion in 2010 to US$247.02 billion in 2024, while pollination-attributable crop output increased from 203.92 million tonnes to 274.73 million tonnes. These results indicate that insect-mediated pollination is not a marginal ecological contribution, but a substantial component of high-value crop production.

This finding is consistent with global assessments showing that animal pollination supports a large share of fruit, vegetable, oilseed, nut and seed crop production [1,2,3]. It also extends earlier Chinese valuation studies, which demonstrated the economic importance of bee or insect pollination but were mostly based on static benchmark years [6,7]. By constructing a 2010–2024 crop-level panel, this study shows that pollination value in China is not only large in absolute terms, but has also increased dynamically alongside agricultural structural change.

The increase in pollination value reflects both economic and agronomic processes. On the economic side, fruits, vegetables and cash and industrial crops have become increasingly valuable within China’s crop production system. On the agronomic side, many of these crops show moderate, high or essential dependence on insect pollination. The combination of high production value and biological dependence means that pollination services are increasingly embedded in China’s high-value agricultural production. This supports the argument that pollination should be treated as a production-related input rather than only as an external ecosystem service.

The implications of this increasing pollination value extend beyond monetary production value. Pollinator-dependent crops are also important sources of micronutrients, including vitamin A, vitamin C, folate and vitamin E. Previous studies have shown that pollinator-mediated crops contribute disproportionately to nutrient supply and that pollinator deficits may affect food consumption and human health [28,29]. Although the present study did not directly estimate dietary intake, the dominance of fruits and vegetables in pollination service value suggests that the widening pollination gap may also have implications for the production-side supply of nutrient-rich foods.

### 4.2. Relative Vulnerability Reveals Structural Exposure in Horticultural Production

A key contribution of this study is the incorporation of relative vulnerability. Absolute pollination service value measures the economic value attributable to pollination, whereas relative vulnerability measures the share of crop value exposed to pollination dependence. These two indicators are related but not equivalent.

The results show that national RVmid remained high, increasing from 35.91% in 2010 to 36.94% in 2024. This indicates that more than one-third of the gross production value of the selected crops was exposed to pollination dependence under the benchmark coefficient. Although absolute pollination value increased substantially, national RV changed only moderately. This suggests that the rise in pollination value was driven mainly by the expansion of high-value crop production rather than by a dramatic shift in aggregate dependence coefficients.

The category-level results further show that vulnerability was concentrated in horticultural crops. In 2024, fruit crops had the highest RVmid, at 50.20%, followed by vegetable crops at 46.73%. By contrast, cash and industrial crops and staple and field food crops showed much lower vulnerability, at 13.66% and 5.05%, respectively. This pattern implies that pollination risk is unevenly distributed across the agricultural system. Staple-oriented crop categories are relatively less exposed, whereas fruit and vegetable systems are proportionally more dependent on pollination services.

This distinction matters for risk assessment. Vegetable crops contributed the largest absolute pollination service value, while fruit crops had the highest proportional vulnerability. Thus, economic exposure and proportional vulnerability identify different aspects of pollination risk. Policy prioritization should therefore avoid relying on a single indicator. Absolute value identifies where potential economic losses are largest, while RV identifies where crop production is most structurally exposed to pollination dependence.

### 4.3. Managed Honeybee Supply Remains Far Below Theoretical Pollination Demand

The supply–demand analysis reveals a large structural gap between theoretical honeybee pollination demand and managed colony supply. In 2024, adjusted honeybee pollination demand reached 73.73 million colonies, whereas the observed managed honeybee colony stock was only 9.54 million colonies. Even before applying the effective supply correction, theoretical demand was more than seven times the nominal colony stock.

After applying the effective availability coefficient, the imbalance became more pronounced. Effective colony supply was estimated at 5.73 million colonies in 2024, producing an effective supply gap of 68.00 million colonies and an effective demand-to-supply ratio of 12.88. The effective availability coefficient was introduced to distinguish nominal colony stock from effective pollination-service capacity. Nominal colony stock includes all registered managed colonies, but not all colonies are strong enough, geographically matched to crop-flowering areas, available during crop flowering periods, or economically mobilized for commercial crop pollination. Therefore, θ = 0.60 should be interpreted as a benchmark correction for effective availability rather than as an empirically observed national share of colonies used for pollination services. This indicates that nominal colony stock may substantially overstate effective pollination capacity when not all colonies can be mobilized for crop pollination. This also means that increasing effective pollination supply is not merely a matter of moving more colonies. Migratory beekeeping may influence pathogen prevalence and dispersal between managed and wild bees [30], so policies aimed at reducing pollination deficits should also incorporate colony-health monitoring and biosecurity measures.

This finding is consistent with international evidence that managed honeybee stocks may not be sufficient to satisfy the growing demand for crop pollination [8,9,10]. Aizen and Harder [8] showed that the global stock of domesticated honeybee hives has grown more slowly than agricultural demand for pollination. Breeze et al. [9] identified similar supply–demand mismatches in Europe. Mashilingi et al. [10] further estimated global honeybee pollination demand using harvested area and recommended colony densities and concluded that managed honeybees are insufficient to provide optimal pollination services worldwide. The Chinese case presented here adds national-scale evidence that even a large beekeeping sector may not be sufficient if colony supply is evaluated against theoretical crop-level demand.

The estimated gap should be interpreted as a theoretical supply-capacity gap rather than direct evidence of realized pollination failure. Actual crop pollination may be partly supported by wild pollinators, other managed insects, wind pollination, self-pollination, manual pollination or suboptimal pollination that still produces some yield. Nevertheless, the magnitude of the gap indicates that China’s current managed honeybee stock is unlikely to satisfy optimal stocking requirements for all pollination-responsive crops. This suggests that the beekeeping sector has not yet been fully developed as a pollination-service infrastructure for high-value agriculture.

### 4.4. Value, Vulnerability and Colony Demand Identify Different Crop Priorities

The crop-level results show that pollination value, relative vulnerability and colony demand identify different dimensions of risk. The crops contributing most to pollination service value were not necessarily those generating the largest honeybee colony demand.

In 2024, the largest contributors to pollination service value were mainly cucurbits and fruits, including cucumbers and gherkins, apples, watermelons, pears, and tangerines, mandarins and clementines. These crops combine high production value with high or essential dependence on insect pollination, and therefore represent major economic exposure to pollination disruption.

By contrast, the largest contributors to colony demand included rape or colza seed, soybeans, groundnuts, tangerines, mandarins and clementines, and apples. Some of these crops, especially rape or colza seed and soybeans, generated high colony demand because of extensive harvested area, even though their relative vulnerability or absolute pollination service value was lower than that of major cucurbits and fruits.

This divergence has important implications for pollination management. Economic valuation identifies where pollination loss may cause the greatest monetary impact, while colony-demand analysis identifies where the largest number of colonies would be required to meet recommended stocking densities. A crop such as cucumber may represent high economic value at stake, whereas a crop such as rapeseed may represent high physical demand for colony allocation. Therefore, pollination policy should differentiate among crop types: high-value horticultural crops require stable commercial pollination services and pesticide-risk control, while large-area cash and industrial crops require regional colony-allocation planning and landscape-scale coordination.

The effective supply adjustment also highlights that nominal colony stock should not be equated with effective pollination capacity. Colonies may be used primarily for honey production, located far from crop-demand areas, constrained by migration routes, weakened by disease or pesticide exposure, or excluded from crop fields because pollination-service fees are insufficient. In this study, the benchmark decomposition attributed 2.29 million effective colonies to crop nectar-source support and 3.44 million colonies to wild or semi-natural nectar-source support in 2024. This distinction emphasizes that crop flowering resources and non-crop floral resources play different roles: crop flowering generates concentrated commercial pollination demand, whereas wild and semi-natural floral resources support colony maintenance, recovery and seasonal continuity.

### 4.5. Implications for Pollination-Service Markets and Agricultural Risk Governance

The findings suggest that China’s beekeeping sector should be understood not only as a bee-product industry, but also as a strategic biological input system for high-value agriculture. Current agricultural policy and market institutions still tend to emphasize honey and other bee products, while the crop pollination function of managed honeybees remains underdeveloped. This institutional imbalance may partly explain why nominal colony stock does not translate into sufficient effective pollination capacity.

A more explicit pollination-service market is needed. Formal pollination contracts, recommended service fees, insurance mechanisms and local coordination platforms could reduce transaction costs between growers and beekeepers. Without adequate compensation, beekeepers have weak incentives to enter crop systems where honey production is limited, pesticide exposure is high or pollination service income is uncertain.

Pesticide governance during flowering periods is also essential. Poorly timed pesticide applications can reduce colony survival, weaken colony strength and discourage beekeepers from entering crop-producing areas. This issue is becoming more urgent with the spread of mechanized and drone-based spraying. Flowering-period restrictions, advance notification systems and coordination among growers, pesticide applicators and beekeepers would help reduce avoidable pollinator exposure. This is consistent with evidence that pesticide exposure, parasites and lack of floral resources can jointly drive bee declines and reduce pollination service delivery [31,32,33].

The results also point to the need for crop-specific strategies. High-value and high-vulnerability crops, such as cucumbers, watermelons, apples, pears and citrus-type fruits, should be prioritized for stable commercial pollination services and pesticide-risk reduction. Large-area crops with high colony demand, such as rapeseed, soybeans, groundnuts and cotton, require regional colony-deployment planning. In addition, wild and semi-natural floral resources should be incorporated into pollination policy because managed honeybees require continuous resources beyond short crop-flowering periods. Maintaining field margins, flowering strips, grasslands, diversified orchard groundcover and semi-natural habitats can support colony health and improve effective pollination capacity.

### 4.6. Limitations and Future Research

Several limitations should be acknowledged. First, pollination-dependence coefficients were assigned using literature-based crop-level ranges. Although the use of $D_{low}$, $D_{mid}$, and $D_{high}$ provides an uncertainty interval, actual dependence may vary by cultivar, region, management practice, pollinator community and climatic conditions. Future studies should incorporate region-specific and cultivar-specific dependence coefficients where data are available.

Second, recommended colony densities were based on a benchmark crop-category scheme. This approach is suitable for national-scale macro assessment, but it cannot capture local variation in planting density, flowering intensity, field size, colony strength or pollination-management practices. More precise estimates would require crop-specific technical standards and regional agronomic data.

Third, the effective supply coefficient was used as a benchmark correction rather than an empirically observed national parameter. The assumption that 60% of nominal colonies are effectively available for crop pollination is intended to distinguish nominal stock from effective pollination-service capacity. Future work should estimate this parameter using beekeeper surveys, migratory beekeeping records, pollination-service contracts and regional colony-deployment data.

Fourth, the analysis was conducted at the national scale. National-level results may mask regional mismatches between crop pollination demand and colony supply. Provinces with intensive fruit or vegetable production may face more severe local shortages even if national colony numbers appear large. Future research should integrate provincial crop distributions, beekeeper migration routes, flowering calendars, pesticide-risk maps and regional hive-density data.

Finally, this study focused mainly on managed honeybees. Wild pollinators and other managed pollinators may contribute to crop pollination and partially reduce the apparent honeybee supply gap. However, their contribution depends on habitat quality, landscape structure, pesticide exposure and local pollinator diversity. Future research should integrate managed and wild pollination services to evaluate the full pollination portfolio of Chinese agriculture.

## 5. Conclusions

This study demonstrates that China’s agricultural production is increasingly dependent on pollination services, while managed honeybee colony supply remains insufficient relative to theoretical crop pollination demand. From 2010 to 2024, benchmark pollination service value increased from US$114.27 billion to US$247.02 billion, and pollination-attributable crop output increased from 203.92 million tonnes to 274.73 million tonnes. In 2024, 36.94% of the gross production value of selected crops was exposed to pollination dependence, with vegetable and fruit crops contributing more than 93% of total benchmark pollination service value.

The supply–demand results reveal a large structural shortage. Adjusted honeybee pollination demand reached 73.73 million colonies in 2024, compared with a nominal managed colony stock of 9.54 million colonies and an effective supply of only 5.73 million colonies. The resulting effective supply gap was 68.00 million colonies, and theoretical demand was nearly 13 times the effective managed honeybee supply. These findings indicate that China’s beekeeping sector should be regarded not only as a bee-product industry, but also as a strategic biological input system for high-value agriculture. Policies that support commercial pollination markets, reduce pesticide risks, improve colony deployment and maintain floral-resource continuity are needed to strengthen China’s pollination-service capacity.

## Figures and Tables

**Table 1:** Analytical crop categories used in this study.

Analytical Category	Definition	Crop Items
Staple and field food crops	Cereals, pseudocereals, pulses and selected field crops primarily associated with food-security production or broad field-crop systems.	Rice; Buckwheat; Beans, dry; Soya beans
Fruit and nut crops	Pome fruits, stone fruits, citrus fruits, tropical fruits, berries and nut crops.	Apples; Pears; Quinces; Apricots; Cherries; Peaches and nectarines; Plums and sloes; Grapes; Strawberries; Figs; Persimmons; Kiwi fruit; Citrus fruits; Almonds; Walnuts; Avocados; Coconuts; Mangoes; Papayas
Vegetable and cucurbit crops	Cucurbits and fruit vegetables commonly produced for fresh consumption or horticultural markets.	Cantaloupes and other melons; Cucumbers and gherkins; Pumpkins, squash and gourds; Watermelons; Other beans, green; Peas, green; Chillies and peppers; Eggplants; Tomatoes
Cash and industrial crops	Oil, fibre, beverage and other market-oriented crops mainly grown for processing, industrial use or commercial sale.	Seed cotton; Groundnuts; Linseed; Mustard seed; Oil palm fruit; Rape or colza seed; Safflower seed; Sesame seed; Coffee

## Data Availability

The original contributions presented in this study are included in the article material. Further inquiries can be directed to the corresponding author.

## Supplementary Materials

The following supporting information can be downloaded at: https://www.mdpi.com/article/10.3390/insects17070673/s1, Table S1: Crop classification, pollination-dependence coefficients and recommended colony density used in this study.
