# Supplementary material for: Economic Value, Relative Vulnerability and Honeybee Colony Supply–Demand Imbalance in Chinese Agriculture, 2010–2024
Source: Insects. 2026 Jun 27;17(7):673. doi: 10.3390/insects17070673 (PMC13410687; doi:10.3390/insects17070673)
Supplement: Supplementary file 1 [file insects-17-00673-s001.zip › insects-4354928-supplementary.pdf]

Table S1. Crop classification, pollination-dependence coefficients and recommended colony density used in this study.

FAOSTAT crop item	Analytical category	Subcategory	Dependence class	D_low	D_mid	D_high	RCD	Notes
Rice	Staple and field food crops	Cereal	No dependence	0	0	0	0	Excluded from honeybee-pollination demand
Buckwheat	Staple and field food crops	Cereal/pseudocereal	High	0.4	0.65	0.9	1.4	Field crop with high insect-pollination dependence
Beans, dry	Staple and field food crops	Legume	Low	0	0.05	0.1	1.4	Benchmark legume crop density
Soya beans	Staple and field food crops	Legume/oil crop	Moderate	0.1	0.25	0.4	1.4	Retained in the staple and field food crop category to maintain consistency with the original calculation framework
Apples	Fruit and nut crops	Pome fruit	High	0.4	0.65	0.9	4.4	Benchmark fruit crop density

Pears	Fruit and nut crops	Pome fruit	High	0.4	0.65	0.9	4.4	Benchmark fruit crop density
Quinces	Fruit and nut crops	Pome fruit	High	0.4	0.65	0.9	4.4	Benchmark fruit crop density
Apricots	Fruit and nut crops	Stone fruit	High	0.4	0.65	0.9	4.4	Benchmark fruit crop density
Cherries	Fruit and nut crops	Stone fruit	High	0.4	0.65	0.9	4.4	Benchmark fruit crop density
Peaches and nectarines	Fruit and nut crops	Stone fruit	High	0.4	0.65	0.9	4.4	Benchmark fruit crop density
Plums and sloes	Fruit and nut crops	Stone fruit	High	0.4	0.65	0.9	4.4	Benchmark fruit crop density
Grapes	Fruit and nut crops	Berry fruit	Low	0	0.05	0.1	4.4	Benchmark fruit crop density
Kiwi fruit	Fruit and nut crops	Berry fruit	Essential	0.9	0.95	1	4.4	Benchmark fruit crop density
Strawberries	Fruit and nut crops	Berry fruit	Moderate	0.1	0.25	0.4	4.4	Benchmark fruit crop density
Figs	Fruit and nut crops	Fruit	Moderate	0.1	0.25	0.4	4.4	Benchmark fruit crop density
Persimmons	Fruit and nut crops	Fruit	High	0.4	0.65	0.9	4.4	Benchmark fruit crop density
Lemons and limes	Fruit and nut crops	Citrus fruit	Moderate	0.1	0.25	0.4	4.4	Benchmark fruit crop density

Oranges	Fruit and nut crops	Citrus fruit	Moderate	0.1	0.25	0.4	4.4	Benchmark fruit crop density
Other citrus fruit, n.e.c.	Fruit and nut crops	Citrus fruit	High	0.4	0.65	0.9	4.4	Benchmark fruit crop density
Pomelos and grapefruits	Fruit and nut crops	Citrus fruit	High	0.4	0.65	0.9	4.4	Benchmark fruit crop density
Tangerines, mandarins, clementines	Fruit and nut crops	Citrus fruit	High	0.4	0.65	0.9	4.4	Benchmark fruit crop density
Almonds, in shell	Fruit and nut crops	Nut crop	High	0.4	0.65	0.9	4.4	Benchmark nut/fruit crop density
Walnuts, in shell	Fruit and nut crops	Nut crop	Unclear/assigned zero	0	0	0	4.4	Retained as a nut crop, but pollination dependence was assigned zero because of unclear dependence in the adopted classification
Avocados	Fruit and nut crops	Tropical fruit	High	0.4	0.65	0.9	4.4	Benchmark fruit crop density

Coconuts, in shell	Fruit and nut crops	Tropical fruit	Moderate	0.1	0.25	0.4	4.4	Benchmark fruit crop density
Mangoes, guavas and mangosteens	Fruit and nut crops	Tropical fruit	High	0.4	0.65	0.9	4.4	Benchmark fruit crop density
Papayas	Fruit and nut crops	Tropical fruit	Low	0	0.05	0.1	4.4	Benchmark fruit crop density
Cantaloupes and other melons	Vegetable and cucurbit crops	Cucurbit	Essential	0.9	0.95	1	4.3	Benchmark vegetable/cucurbit density
Cucumbers and gherkins	Vegetable and cucurbit crops	Cucurbit	Essential	0.9	0.95	1	4.3	Benchmark vegetable/cucurbit density
Pumpkins, squash and gourds	Vegetable and cucurbit crops	Cucurbit	Essential	0.9	0.95	1	4.3	Benchmark vegetable/cucurbit density
Watermelons	Vegetable and cucurbit crops	Cucurbit	Essential	0.9	0.95	1	4.3	Benchmark vegetable/cucurbit density
Other beans, green	Vegetable and cucurbit crops	Green legume vegetable	Low	0	0.05	0.1	4.3	Benchmark vegetable crop density

Peas, green	Vegetable and cucurbit crops	Green legume vegetable	No dependence	0	0	0	4.3	Included in colony-demand calculation based on vegetable crop density
Chillies and peppers, green (Capsicum spp. and Pimenta spp.)	Vegetable and cucurbit crops	Fruit vegetable	Low	0	0.05	0.1	4.3	Benchmark vegetable crop density
Eggplants (aubergines)	Vegetable and cucurbit crops	Fruit vegetable	Moderate	0.1	0.25	0.4	4.3	Benchmark vegetable crop density
Tomatoes	Vegetable and cucurbit crops	Fruit vegetable	Low	0	0.05	0.1	4.3	Benchmark vegetable crop density
Seed cotton, unginned	Cash and industrial crops	Fibre crop	Moderate	0.1	0.25	0.4	2.5	Benchmark fibre/cash crop density
Groundnuts, excluding shelled	Cash and industrial crops	Oil crop	Low	0	0.05	0.1	2.5	Benchmark oil/cash crop density
Linseed	Cash and industrial crops	Oil crop	Low	0	0.05	0.1	2.5	Benchmark oil/cash crop density
Mustard seed	Cash and industrial crops	Oil crop	Moderate	0.1	0.25	0.4	2.5	Benchmark oil/cash crop density

Oil palm fruit	Cash and industrial crops	Oil crop	Low	0	0.05	0.1	2.5	Benchmark oil/cash crop density
Rape or colza seed	Cash and industrial crops	Oil crop	Moderate	0.1	0.25	0.4	2.5	Benchmark oil/cash crop density
Safflower seed	Cash and industrial crops	Oil crop	Low	0	0.05	0.1	2.5	Benchmark oil/cash crop density
Sesame seed	Cash and industrial crops	Oil crop	Moderate	0.1	0.25	0.4	2.5	Benchmark oil/cash crop density
Coffee, green	Cash and industrial crops	Beverage crop	Moderate	0.1	0.25	0.4	2.5	Benchmark cash and industrial crop density
